# Effects of home visiting programmes on community-dwelling older adults with chronic multimorbidity: a scoping review

**DOI:** 10.1186/s12912-023-01421-7

**Published:** 2023-08-12

**Authors:** Anabel Chica-Pérez, Iria Dobarrio-Sanz, María Dolores Ruiz-Fernández, Matías Correa-Casado, Isabel María Fernández-Medina, José Manuel Hernández-Padilla

**Affiliations:** 1 Almeria Health District, Almeria, 04009 Spain; 2 Department of Nursing, Physiotherapy and Medicine, University of Almeria, 04120 Almeria, Spain

**Keywords:** Community-dwelling, Home visits, Multimorbidity, Nurses, Older adults

## Abstract

**Background:**

Chronic ultimorbidity is the most frequent and serious health problem in older adults. Home visiting programmes could be a strategy with potential benefits. However, there are no scoping reviews to date that examine the effects of home visiting programmes on community-dwelling older adults with chronic multimorbidity.

**Objective:**

To examine the effects of home visiting programmes on community-dwelling older adults with chronic multimorbidity.

**Methods:**

A scoping review was carried out following PRISMA-ScR reporting guidelines. The search was conducted in six databases (PubMed/Medline, Cochrane, CINAHL, Web of Science, Scopus and EMBASE) between October 2021 and April 2022.

**Results:**

Four RCTs with 560 patients were included. The visits were carried out by nurses, nursing students, volunteers, and other healthcare professionals. The interventions varied in the number of visits, frequency, duration of follow-up, and whether or not they were combined with other strategies such as telephone calls. Discrepancies were found in the effects of the interventions on quality of life, self-efficacy, self-rated health, and use and cost of health and social services.

**Conclusion:**

This review shows that home visiting programmes could have potential benefits for older adults with chronic multimorbidity. However, its results have been inconclusive. There is a need for high quality studies involving a larger number of patients, in which home visits are the main intervention.

**Supplementary Information:**

The online version contains supplementary material available at 10.1186/s12912-023-01421-7.

## Introduction

Chronic multimorbidity is the coexistence of two or more long-term conditions with slow progression [1], and is the most common and serious health problem in older adults [2–4]. The prevalence of chronic multimorbidity in community-dwelling adults over 65 years of age is around 70% [5–8]. On a functional level, the presence of chronic multimorbidity is associated with sarcopenia, reduced handgrip strength [8, 9], impaired physical functioning [10] and increased risk of functional limitation [11]. Furthermore, older adults with chronic multimorbidity are at increased risk of pressure ulcers and nutritional imbalance [12, 13]. All this negatively affects the autonomy [14] and quality of life of community-dwelling older adults [15, 16]. Chronic multimorbidity and the physical limitations that it causes are associated with higher levels of stress [17] and depressive symptoms in older adults [18]. Indeed, the presence of chronic multimorbidity triples the risk of depression in older adults [19] and is associated with an increased risk of suicide mortality [20]. Chronic multimorbidity increases the risk of loneliness and social exclusion [21, 22] at the same time as it decreases health-promoting behaviours [23] and social participation [24]. Community-dwelling older adults with chronic multimorbidity have a higher risk of hospitalisation [5, 25, 26], they attend emergency department and outpatient clinics more frequently [27, 28], and incur in higher pharmaceutical expenditure [29, 30]. This leads to an increased burden on healthcare systems and total health costs [5, 6, 28, 30, 31].

The organisation of healthcare systems often requires older adults with chronic multimorbidity to see several specialists who treat their health problems in a fragmented way [32–34]. As a consequence, older adults are confronted with complex therapeutic regimens with long lists of medication [33], restrictive dietary indications and drastic changes in lifestyle habits [35]. Therefore, it is important for nurses to implement interventions that help older people with chronic multimorbidity to navigate healthcare systems and foster their self-care and autonomy to manage therapeutic regimens effectively [36–39]. In this regard, the WHO suggests that home visiting programmes could improve the health of community-dwelling older adults with chronic multimorbidity [40, 41]. A home visit is a service in which trained healthcare professionals visit individuals in their own home with the aim of increasing autonomy through primary, secondary and tertiary prevention activities [42]. The effects of home visiting programmes in older people with chronic heart failure [43, 44] and chronic high blood pressure have been studied [45], and are known to be associated with significantly lower mortality [46]. Even those home visiting programmes based on telemedicine have been able to demonstrate improvements in quality of life, self-efficacy and depression levels [47]. However, the available evidence on the effects of home visiting programmes may be contradictory and more research is needed before they can be recommended [42, 48, 49]. Furthermore, after an exhaustive literature search, no literature review has been found that provides evidence on the effects of home visiting programmes on community-dwelling older adults with chronic multimorbidity. Therefore, the aim of this study is to examine the effects of home visiting programmes on community-dwelling older adults with chronic multimorbidity.

## Methodology

### Design

A scoping review of intervention studies was conducted following international guidelines and recommendations [50, 51], as well as the PRISMA-ScR recommendations for reporting results of scoping reviews [52].

### Search strategy

An exhaustive search on six databases (PubMed/Medline, Cochrane, CINAHL, Web of Science, Scopus and EMBASE) was carried out between October 2021 and April 2022. The complete search strategy used on PubMed was: (elderly OR older adults OR aged OR older) AND (multiple AND health AND conditions OR multimorbid OR multimorbidity OR non-communicable disease OR NCD OR chronic disease OR chronic condition) AND (intervention OR program OR programme OR visit* programme OR home visit* OR home visiting OR home based) AND (nurses, visiting OR home visits OR nurs* students OR human volunteers OR trained volunt*). For the rest of the databases, a similar strategy was used with the necessary adaptations (see Additional file 1). Following the PRISMA-ScR’s recommendations [52], we used the PICO approach to formulate our research question: [P] community-dwelling older adults with multimorbidity; [I] home visiting programmes; [C] usual care; [O] health-related outcomes. The question was: “What are the effects of home visiting programmes on health-related outcomes of community-dwelling older adults with chronic multimorbidity when compare to usual care?”

### Eligibility criteria

The following inclusion criteria were considered for study selection: (1) community-dwelling older adults (aged 60 years or older) with chronic multimorbidity; (2) interventional studies: quasi-experimental or randomised clinical trial (RCT); (3) publication in English or Spanish; (4) home visiting programmes based on individual visits. The exclusion criteria were: (1) participants were cognitively impaired; (2) the intervention was focused on a chronic condition; (3) the intervention was a single, isolated home visit. No time limit was set for the results.

### Search results

In the identification phase, searching on the various databases yielded 1199 results. After checking and manually removing duplicate articles (*n* = 203), there were 996 records left. We then eliminated 940 studies after reading the title and abstract. In the eligibility phase, two reviewers from the research team independently assessed 56 full-text articles and conducted an additional search through their references. No new eligible articles were found, and 52 records were discarded for not meeting the eligibility criteria. Four articles were ultimately included. The flowchart shows this article selection process (see Additional file 2).

### Quality assessment and risk of bias

**Fig. 1:**
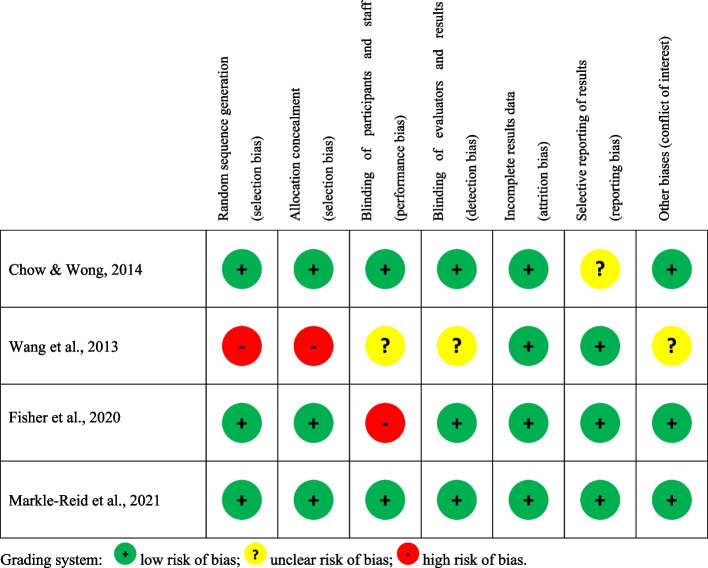
Summary of bias assessment of the included studies [53–56]

The recommendations in the Cochrane Handbook for assessing risk of bias in studies were followed [51]. Two reviewers assessed the quality of the included studies independently, and their assessments were then cross-checked by the senior researcher on the study. To assess the studies’ risk of bias, each criterion was rated as high, low or unclear (Fig. 1).

### Data abstraction and synthesis

Two researchers independently extracted information from the four studies included in this review. Following international guidelines and recommendations [50, 51], a template with the following headings was created: first author's name, year of publication, study title, sample size, study location, study design, intervention characteristics, outcome measures and main findings. The data extraction method was tested with one of the included articles. Neither reviewer reported any differences or problems regarding data extraction. The method was repeated for the rest of the studies. A third experienced reviewer checked the extracted data for accuracy and integrity. A summary of the extracted data can be found in Table 1. A meta-analysis was not possible due to the heterogeneity of the interventions, assessment methods and findings reported in the studies. Therefore, a narrative synthesis of the findings was conducted based on the characteristics of the interventions, the variables studied, and the outcomes reported.

**Table 1:** Summary of results

	**Location**	**Sample size**	**Sample**	**Type of intervention**	**Who carried it out**	**Nº of visits and duration**	**Study time**	**Variables**	**Results**
Chow and Wong, (2014) [53]	Hong Kong	*N* = 312 **IG** HVG: (*n* = 87) PCG: (*n* = 96) **CG** (*n* = 98)	Average age (years): 76.5 80.4% with 2 chronic conditions ♀: 52.5%	**IG (2 arms):** Pre-discharge assessment + in **HVG**: 2 HV (week 1 and 3) + 2 phone calls (week 2 and 4) in **PCG**: 4 phone calls **CG:** routine care + 2 placebo calls (social – 5 min)	**HVG:** **1st visit:** CMN + NS (week 1, 72 h post-discharge) 1st call: CMN (week 2) 2nd visit: NS (week 3) 2nd call: CMN (week 4) **PCG:** 1st: CMN 2nd: NS 3rd: NS 4th: CMN	**Nº visits**: 2 every 15 days in HVG **Duration**: Not recorded	4 weeks **Follow-up**: 12 weeks post-discharge (8 weeks post-intervention)	**Primary results:** *Re-admission rate* (administrative records) **Secondary results:** *Health-related quality of life* (HRQOL) (SF-36). Physical functioning (PF) and psychological functioning (PSYF) *Self-efficacy* Chronic conditions (Chinese version) *Self-assessed health*	**Primary results:** *Re-admission rate*: At 4 weeks: no significant differences At 12 weeks: HVG = 33%, PCG = 28.3%, CG = 45.4% IG vs. CG (× 2 = 8.03; *p* = 0.018); PCG vs. CG (× 2 = 7.25; *p* = 0.007) **Secondary results:** *Quality of life (HRQoL)*: Physical component: HVG vs. PCG vs. CG at 3 time points [F(2, 277) = 4.31; *p* = 0.014]; PCG vs. CG [F(2, 277) = 4.31; *p* = 0.016] At 4 and 12 weeks: PCG vs. CG in: physical functioning ([F = 7.35; *p* = 0.006]; [F = 19.8; *p* < 0.001]); physical role ([F = 4.70; *p* = 0.015]; [F = 11.2; *p* < 0.001]); vitality ([F=6.62; *p* < 0.001]; [F = 8.05; *p* < 0.001]); mental health ([F = 7.08; *p* < 0.001]; [F = 4.29; *p* < 0.033]); difference at 4 weeks: HVG vs PCG in: physical functioning [F = 7.35; *p<*0.001]; vitality [F = 6.62; *p* < 0.040]; social functioning [F = 3.70; *p* = 0.032] At 12 weeks: HVG vs CG in: physical role [F = 11.2; *p* < 0.001]; emotional role [F = 4.08; *p* < 0.013]; mental health [F = 4.29; *p*=0.040] *Self-efficacy*: Within-group effects over time: HVG [F(2, 172)=606; *p* = 0.002], PCG [F(18, 171)=843; *p* < 0.001] At 4 and 12 weeks: PCG vs. CG ([F = 5.1; *p* = 0.005]; [F = 5.39; *p* = 0.031]) At 12 weeks: HVG vs. CG [F = 5.39; *p* = 0.007] *Self-assessed health*: After 4 and 12 weeks: PCG vs. CG ([F = 19.3; *p<*0.001]; [F = 8.67; *p<*0.001])
Wang et al. (2013) [54]	Taiwan	*N* = 62 **IG** (*n* = 30) **CG** (*n* = 32)	Average age (years): 71.3 ≥ 2 chronic conditions ♀: 55%	**IG:** Visit to Primary care + 3 HV (medication safety training) + calls (reinforce medication adherence in 2 months) + Visit to Primary Care **CG:** Routine care	11 volunteers (with training certificate for health care volunteers)	**Nº visits**: 3 HV in weeks 2, 5 and 8 **Duration:** *1st visit:* 2 h aprox *2nd y 3rd visit:* 1 h aprox Call: 20–30 min	2 months (10 weeks) **Follow-up**: not recorded	Questionnaire KAB-MS: *Medication safety knowledge, attitudes and behaviours*	*Knowledge*: IG vs. CG (*p*=0.012); and within-group IG (*p* < 0.001) *Attitudes*: IG vs. CG no significant differences *Behaviours*: IG vs. CG: significant differences in 3 safety behaviours: - When they receive prescription (*p* = 0.013) - Before taking them (*p* = 0.003) - Surplus care (*p* = 0.025)
Fisher et al. (2020) [55]	Ontario, Canada	*N*=32 **IG** (*n* = 16) **CG** (*n* = 16) *ITT* *N* = 59 **IG** (*n* = 30) **CG** (*n* = 29)	Average age: not recorded ≥ 3 chronic conditions (x̄ 8.6 IG, 8.7 CG) ♀:49%	**IG:** HV by interprofessional groups + monthly conferences (in collaboration with patient and carer/family member) + Care management ** + **Routine care **IG:** Routine home care (without interprofessional team)	Interdisciplinary team.: CC, NUR, PT, OT y PSW	**Nº visits**: min. 1 of PSW, min 1 of CC and min. 3 of PT o OT* *according to patients, possible substitution. 1 CC for a PT or 2 NUR **min 5-max 16**	6 months **Follow-up**: not recorded	**Primary results:** *Quality of life* (HRQoL) (PF SF-12) **Secondary results:** *Mental functioning* (PSYF SF-12) *Depressive symptoms* (CESD-10) *Anxiety* (GAD-7) *Self-efficacy* (Chronic conditions) *Use of medical care (cost)* (HSSUI)	**Primary results:** HRQoL: IG vs CG in *general health* [Mean difference = 8.72 (95% CI =2.3–15.14); *p* = 0.01] **Secondary results:** no significant differences in *mental functioning, depressive symptoms, anxiety or self-efficacy* *Use of medical care*: Hospitalization*:* within-group IG ( *p* =0.01) Visits to emergency services*:* within-group CG (*p* = 0.02) Costs*:* in home care. In favour of CG (z=-3.00; *p* = 0.003)
Markle-Reid et al (2021) [56]	Ontario, Canada	*N* = 99 **IG** (*n* = 47) **CG** (*n* = 52) *ITT* *N* = 127 **IG** (*n* = 63) **CG** (*n* = 64)	Average age: 77 years 90% ≥ 6 chronic conditions. (x̄ de 8) ♀:63%	**IG:** - HV (max. 6, min. 2) + calls + accompaniment by Healthcare System **CG:** Routine home care	NUR (care transition coordinator)	**Nº visits**: max. 6, min. 2* (Average of 3) **Duration**: each visit 1 h approx *45 received at least 1 h	6 months **Follow-up**: At 12 months (6 months after ending intervention)	**Primary results:** *Mental functioning* (PSYF VR-12) **Secondary results:** *Physical functioning* (PF VR-12) *Depressive symptoms* (CESD-10) *Anxiety* (GAD-7) *Perceived social support* *Patient experience* (CCCQ e IC-PREM) *Self-efficacy* (Chronic conditions) *Use of medical care (costs)* (HSSUI)	**Primary results:** no significant differences **Secondary results:** no significant differences in *physical functioning, depressive symptoms, anxiety or perceived social support* *Patient experience*: IG vs CG in received information (× 2 = 4.88; *p* = 0.03) *Use of medical care*: in home care. In favour of CG (z=7.14; *p* < 0.001)

*CC* Care coordinator, *PF* Physical functioning, *PSYF* Psychological functioning, *HRQoL* Health-related quality of life, *NUR* Nurse, *CMN* Case manager nurse, *NS* Nursing student, *PT* Physiotherapist, *CG* Control group, *IG* Intervention group, *PCG* Phone call group, *HVG* Home visiting group, *PSW* Personal support worker, *OT* Occupational therapist, *T* Time, *HV* Home visit, *vs.* versus, *x̄* media

## Results

### Narrative summary

This scoping review included 4 studies with 532 community-dwelling older adults with chronic multimorbidity. All studies were randomised clinical trials (RCT) [53–56], and involved the implementation of a home visiting programme. The interventions varied widely in terms of who conducted them (nurse case managers in collaboration with nursing students, volunteers instructed by nurses, interprofessional team, and nurses), their characteristics (follow-up calls, number of visits, duration and follow-up), sample size and reported outcomes (see Table 1). The studies were conducted from 2012 to 2018 in Hong Kong [53], Taiwan [54] and Canada [55, 56], and they were published between 2013 and 2021.

### Characteristics of the interventions

Interventions were delivered by case manager nurses (CMNs) assisted by nursing students (*n* = 1) [53], volunteers instructed by nurses (*n* = 1) [54], an interprofessional team (care coordinator (CC), nurse, physiotherapist, occupational therapist and a personal support worker) (*n* = 1) [55], and nurses who acted as care coordinators (*n* = 1) [56]. All of the people responsible for delivering the interventions in each of the studies had attended prior workshops or training programmes. The total number of older adults who participated in each study was 312 [53], 62 [54], 59 [55], and 127 [56]. The interventions lasted either 4 weeks (*n* = 1) [53], 2 months (*n* = 1) [54] or 6 months (*n* = 2) [55, 56]. The number of home visits carried out ranged from a minimum of 2 [56] to a maximum of 16 [55]. The duration of the visits was not reported in half of the studies [53, 55] and in the remaining two, it ranged from one to two hours [54, 56]. Three of the included studies supplemented the home visiting programme with telephone calls [53, 54, 56]. The fourth study relied only on home visits [55]. After completing the intervention, there was a follow-up in two of the four studies, ranging from 8 weeks [53] to 6 months [56].

The study by Chow & Wong (2014) had three arms and two intervention groups [53]. The first intervention group received home visits that alternated with telephone calls on a weekly basis for a total of four weeks (HVG). The second intervention group received solely telephone calls (PCG). Participants involved in both interventions received a pre-discharge assessment based on the Omaha System [57]. In the first intervention, the home visits were conducted by student nurses, who were supervised by a case manager nurse (CMN) only on the first visit. The students received prior information about each patient’s condition as well as six hours of training in communication, education and multimorbidity management. In the second intervention, the CMN made the first and fourth phone calls. The student nurses made the second and third calls, continuing with the interventions to meet the agreed objectives. Meanwhile, the control group (CG) received two social phone calls that lasted approximately five minutes and that were four weeks apart.

In the study by Wang et al. (2013), [54] a visiting programme was conducted to improve the medication safety of older adults in a rural area and compared to usual care. The intervention was carried out by volunteers who received 26 hours of pre-training by nurses and other members of the Primary Health Care (PHC) team, where they became proficient in the use of the Medication Safety Guide (MSG). The home visits varied in duration and content. During the first visit, the volunteer gave each participant a MSG, checked the prescribed medication and used stickers on each container to identify the shape, colour and number of pills, as well as the schedule. The second and third home visits were motivational in nature with the aim of encouraging medication adherence and safety. In addition, participants had to demonstrate what they had learned during the first visit.

In the study by Markle-Reid et al. (2021), [56] a hospital-to-home transitional care intervention comprising home visits, phone calls and accompaniment through the health system was conducted to assess its effectiveness compared to usual care. One of the inclusion criteria, in addition to age and multimorbidity, was that participants had to be positive for depressive symptoms according to the Patient Health Questionnaire (PHQ-2) [58]. The number of phone calls and home visits was not pre-set. Participants could receive monthly home visits by the nurse practitioners. Participants were called based on the number of home visits made. The home visits and phone calls included conducting a comprehensive assessment of participants’ needs, promoting multimorbidity management and medication adherence, and promoting increased social participation.

In the study conducted by Fisher et al. (2020), [55] no telephone calls were made alongside visits, and the intervention was carried out by an interprofessional team. All members conducted home visits individually. The schedule, team configuration, care plan, number of visits and who conducted the visits varied according to the budget and the participant’s needs and preferences. Each case was discussed in at least three conferences. In addition, the CCs carried out ongoing case management, which facilitated access and communication with health and social services. The CG received regular home care services.

Table 1 shows more details about the studies included in this review.

### Participants’ outcomes

#### Quality of life

Health-related quality of life (HRQoL) was assessed in three of the four studies [53, 55, 56]. In the Chow & Wong (2014) [53] study, HRQoL was assessed using the SF-36 health questionnaire at baseline, at 4 weeks and at 12 weeks after the start of the intervention. After the 4-week intervention, the PCG scored significantly higher than the CG for the domains “physical functioning", “physical role”, “vitality” and “mental health”. Furthermore, significant improvements were also found in “physical functioning”, “vitality” and “social functioning” for the HVG compared to the PCG. At 12 weeks, the PCG showed significant improvements in “physical functioning”, “physical role”, “vitality” and “mental health” when compared to the CG. Significant improvements were also found in the HVG compared to the CG in “physical role”, “emotional role”, and “mental health”. No significant differences were found between IGs at 12 weeks. In the work of Fisher et al. (2020), [55] the SF-12 questionnaire was used to assess HRQoL, finding significant improvements only in the domain of “general health” in favour of the IG after 6 months of intervention. In Markle-Reid et al. (2021), [56] physical and mental functioning were assessed with the Veterans RAND 12 Item Health Survey (VR-12), with no statistically significant differences found between the intervention group (IG) and CG from the baseline to the end of the 6-month intervention.

#### Self-efficacy in managing chronic conditions

Two studies assessed self-efficacy in managing chronic conditions [53, 55]. In the study by Chow & Wong (2014) [53], the Chinese version of the Stanford Self-Efficacy Scale for Chronic Disease Management was used. Self-efficacy was assessed at baseline, at 4 weeks and at 12 weeks after the start of the intervention. For within-group effects, both the HVG and the PCG demonstrated significant improvements over time. At 4 weeks, significantly higher self-efficacy was found for PCG compared to the CG. At 12 weeks, participants in both the HVG and the PCG showed significantly higher self-efficacy levels than those allocated to the CG. The study by Fisher et al. (2020) [55] used the original version of the Stanford Self-Efficacy Scale for Chronic Disease Management. No significant differences were found between groups after 6 months of intervention.

#### Self-rated health

Self-rated health was only assessed in the Chow & Wong (2014) [53] study using a single-item Likert scale ranging from excellent to poor. At 4 and 12 weeks, participants in the PCG scored significantly higher than those in the CG.

#### Use and cost of health and social services

Two studies evaluated the effects of interventions on the use of health and social services [53, 55]. The study by Chow & Wong (2014) [53] assessed the rate of unplanned hospital readmissions at 28 and 84 days post-discharge using the hospital’s administrative record system. At 28 days post-discharge, although the HVG and PCG had lower absolute readmission rates than the CG, no significant differences were found between the groups. However, at 84 days post-discharge, lower readmission rates were found in the HVG and PCG compared to the CG. In the study by Fisher et al. (2020), [55] the use of health and social services was assessed using the Health and Social Services Utilisation Inventory (HSSUI). The rate of hospitalisation was assessed, and no statistically significant differences were found between the IG and CG. In addition to the hospitalisation rate, they measured the effect of the intervention on the number of visits to the emergency department, finding no significant differences between the IG and CG after 6 months of the intervention.

Two studies measured the effect of interventions on health service costs [55, 56]. Fisher et al. (2020) [55] found no significant differences between groups in total cost from baseline to 6 months. Significantly higher costs were found for home and outpatient care after 6 months of the intervention compared to the CG. The study by Markle-Reid et al. (2021) [56] also found no significant differences between groups for total cost after 6 months of intervention. The authors found that the IG incurred significantly higher costs associated with home visiting and training.

#### Other results

In the work of Wang et al. (2013), [54] knowledge, attitude and behaviour in relation to medication safety were assessed using the KAB-MS questionnaire. Significantly higher scores on medication safety knowledge were found for the IG compared to the CG. Regarding medication safety behaviours, significant differences were found in favour of the IG in checking medication when receiving the prescription, in checking medication before taking it and in taking proper care of surplus medication.

Two studies assessed depressive symptoms, anxiety and mental functioning [55, 56]. Both studies used the Centre for Epidemiological Studies Depression Scale (CES-D-10) to assess depressive symptoms. For anxiety, they used the Generalised Anxiety Disorder Scale (GAD-7). Mental functioning was assessed through different scales. In the study by Fisher et al. (2020), [55] it was assessed through the mental component of the SF-12 questionnaire. In Markle-Reid et al.’s (2021) [56] study, it was assessed through the mental component of the VR-12. Neither of the studies found significant differences in depressive symptoms or anxiety [55, 56].

Perceived social support was assessed in the study by Markle-Reid et al. (2021) [56] using the Personal Resources Questionnaire (PRQ2000). No significant differences were found between groups. This same study also assessed patient satisfaction with the care received. Satisfaction with care was measured using the Client-Centred Care Questionnaire (CCCQ) and Patient-Reported Experience Measures of Integrated Care at Home for Older People (IC-PREMs). No significant differences were found between groups except for the item related to obtaining information about health and social services, for which the IG reported receiving significantly more information than the CG.

## Discussion

This scoping review of four studies examined the effects of home visiting programmes on community-dwelling older adults with chronic multimorbidity. The interventions varied in terms of the providers, sample size, duration, frequency and content. Home visiting programmes for community-dwelling chronically multimorbid older adults, in which home visits are the only intervention, have not been studied extensively. In fact, most of the studies included in this review complement home visits with telephone calls [53, 54, 56].

HRQoL was explored in two of the studies included in our review [53, 55]. HRQoL improved significantly immediately after the intervention in the PCG, and after the follow-up in the HVG [53]. Fisher et al. (2020) [55] also found significant differences after the intervention in the general health domain, which coincides with a study involving patients with Diabetes Mellitus and other associated morbidities [59]. While Chow & Wong (2014) [54] used the SF-36 to assess HRQoL, the other studies used the SF-12. The lack of significant improvements in HRQoL could be associated with the sensitivity of the different questionnaires used to assess it, and differences in patients’ perceptions [60]. Furthermore, according to Markle-Reid et al. (2018), [59] a longer follow-up of the effects of interventions may be necessary to see an effect on quality of life. In addition, the majority of studies in this review included more women than men and this could also explain why HRQoL has not improved across the board, since men respond better to self-care interventions involving education and support from others [61].

Self-efficacy in chronic disease management was found to improve immediately after the intervention for the PCG and at follow-up for both the PCG and the HVG [53]. This could imply that the combination of face-to-face visits and calls are more likely to achieve greater benefits and have long-term effects on both HRQoL and self-efficacy [62–64]. Fisher et al. (2020) [55] and Markle-Reid (2018) [59] did not find that home visits significantly improved self-efficacy amongst older adults with chronic multimorbidity. These results differ from the evidence showing that a psychological home visiting programme can improve self-efficacy in older adults with a chronic condition [65]. According to Hur (2018) [66], socio-economic factors influence self-efficacy and are therefore more likely to have an impact on older adults. Since in the study by Fisher et al. (2020) [55] the visits were set according to the participants’ budget and preferences, this could have influenced the results.

Chow & Wong’s study (2014) [53] found improvements in self-assessed health in the PCG but not in the HVG. This is likely to be because phone calls alone have positive effects on self-assessed health without the need for visits [67]. Therefore, the positive effects on self-assessed health found in other studies that combined visits and calls could have been achieved with calls alone [62, 63]. While calls could achieve immediate improvements after the intervention, visits could contribute to maintaining the effects over time [53]. However, contrary to these hypotheses, improvements in self-rated health have also been seen through home visits alone [68]. The feasibility of a virtual visiting programme, a hybrid modality between face-to-face visits and telephone calls for community-dwelling older adults with multimorbidity could be questioned [69–72]. Nonetheless, face-to-face communication is known to be more beneficial than telematic communication for older adults [73].

In terms of the effects of home visiting programmes on the use of health services, the absolute number of readmissions was lower in the intervention groups [53, 55]. However, the results of these studies are not consistent and concur with the evidence showing that while in some contexts home visits are effective to reduce hospital admissions [46], in other contexts they are not [74, 75]. These differences could be explained by the fact that face-to-face visit are considered the ideal way to detect and manage symptoms early, thus preventing relapses and readmissions [76]. In the studies included in this review, the interventions were not found to achieve a significant reduction in the frequency of ED visits [55]. Nonetheless, a significant reduction in frequency over time was found within the CG, which could be explained by the fact that the healthcare professionals conducting the home visits were probably able to resolve the situation without the need for referral to the emergency department [77].

The studies that explored expenditure on health services showed no significant differences for total costs [55, 56]. This is in line with the study by Seidl et al. (2015), [62] where no significant differences in total cost were found either. However, significant increased costs related to home and outpatient care for the intervention groups were found [55, 56]. This could be because home visits combined with calls improve an individual’s ability to recognise symptoms [78], which may prompt them to seek help and thus increase health spending [79].

Knowledge, attitude, and behaviour in relation to medicine safety were only assessed in the study by Wang et al. (2013), [54] whose intervention only resulted in significant changes in knowledge and some behaviours (checking medication when receiving prescription; checking medication before taking them; correct care of surplus medication). These results could be due to the complex process required to change one’s attitude, as it is necessary to address the various factors that make up an individual's personality in order to achieve this change [80]. A study exploring patients with hypertension who live in poverty had similar results; a nurse-led home visiting programme achieved significant improvements in understanding and controlling hypertension, as well as in managing the therapeutic regimen [45]. However, a low socioeconomic and educational level are known to have a negative impact on adherence to the treatment regimen [81]. This may have required greater precision and complexity in the interventions, even more so for patients with multimorbidity and associated polypharmacy [82], which may have favoured positive results.

Although evidence suggests that home visits can improve mental health [62, 83], none of the studies included in this review found significant differences between home visits and other interventions [55, 56]. This may be because depressive symptoms in people with multimorbidity overlap with somatic symptoms, often confounding the results [84]. In fact, in Markle-Reid et al. (2021) [56], participants were selected if they had depressive symptoms. This may have overlapped with manifestations of multimorbidity that are highly difficult to reverse [84, 85], and may explain why their intervention did not improve perceived social support [86]. Another aspect assessed in the Markle-Reid et al. (2021) [56] study was the success of the item on obtaining information about health and social services. This result is related to the fact that part of the intervention consisted of explaining how health and social services work. Similarly, in a study in which telephone calls were conducted, positive effects were reported in relation to the information received [87]. This could be due to the quality in the organisation and content of the interventions [88] and that this population has one of the greatest needs for information [89]. However, simply reporting having received information does not guarantee that it is adequate [90].

Although all participants included were adults over 60 years of age, the fact that some studies included people of extreme ages may have influenced the results. Regardless of the number of chronic conditions, it is likely that there was a large difference in functional capacity and health levels between the lower and higher ages. In the context of aging, chronic conditions become more prevalent and common [91, 92], with those living longer being more likely to have experienced better health that allowed them to reach that age [93, 94]. Dividing the sample by age group to present the results could have provided additional information [95]. Another important point to note is that some of the studies required their participants to have at least 2 chronic conditions, while others had at least 3. This may imply significant differences [96, 97], as the number of chronic conditions correlates with the presence of greater complexity and complications [98, 99]. In addition, studies such as Chow & Wong (2014) [53] were limited to a narrow list of chronic conditions. They may have underestimated the presence of chronic conditions in the study’s participants, as well as excluded valid subjects from the sample [100]. In Chow & Wong’s study (2014) [54], home visits were carried out with patients who had recently been discharged from hospital. This situation could have maximised the intervention’s positive effects on the participants [101].

### Limitations

This scoping review has several limitations. The number of studies included is limited, which had an impact on the accuracy of our results. Furthermore, studies with different designs but with relevant data may have been excluded. We excluded studies that did not specify whether all their participants were older adults with chronic multimorbidity, even though they implemented home visiting programmes. On the other hand, one of the included studies evaluated a transition programme and there are already systematic reviews on the effects of this type of intervention. Nevertheless, the intervention was implemented entirely in the participants’ homes, and they all met our study’s inclusion criteria. In addition, we found differences in the way the statistical results were presented in the studies included, both within and between studies. Likewise, it is worth highlighting the studies’ heterogeneity in terms of the healthcare professionals who carried out the visits, the duration of the visits, their frequency, as well as their content. In most of the included studies, home visits are carried out alongside telephone calls; this shows the lack of studies only reporting the effects of a home visiting programme in community-dwelling older adults with chronic multimorbidity. In addition, participants in two of the included studies were recruited immediately after hospital discharge. In this respect, the acute health conditions leading to hospital admission may have influenced the results. Therefore, their comparison with the other studies should be interpreted with caution. It is also important to consider that the settings in which the studies were conducted were very diverse and with certain organisational particularities, so it is difficult to be sure whether the interventions and the results are transferable to other contexts. Similarly, the inclusion of studies that are 10 years old may detract from the relevance of the interventions evaluated in the current global context. The varying quality of the few included articles, as well as the presence of risk of bias in some of them, may limit the validity of this review’s findings.

## Conclusion

Home visiting programmes, combined with other active follow-up strategies such as telephone calls, have the potential to improve some health-related outcomes amongst community-dwelling older adults with chronic multimorbidity. However, the results of this review have been inconclusive. Although improvements in quality of life, self-rated health, rate of readmissions and emergency visits, self-care and self-efficacy have been found, it is difficult to ascertain whether these effects are caused by the visits, the phone calls or a combination of both. Furthermore, the methodological approaches and the way in which the results of some of the studies included in this review have been reported call for a cautious interpretation of the results. There is a need for studies with higher methodological quality, larger sample sizes and that focus exclusively on individualised home visits according to each person’s needs.

### Supplementary Information

**Additional file 1. **Bibliographic/literature search.

**Additional file 2. **Flow diagram adapted from PRISMA-ScR [51].

## Data Availability

All data generated or analysed during this study are included in this published article. Datasets are available through the authors upon reasonable request.
