# Supplementary material for: Effects of home visiting programmes on community-dwelling older adults with chronic multimorbidity: a scoping review
Source: BMC Nurs. 2023 Aug 12;22:266. doi: 10.1186/s12912-023-01421-7 (PMC10422812; doi:10.1186/s12912-023-01421-7)
Supplement: Supplementary file 1 — Additional file 1. Bibliographic/literature search. [file 12912_2023_1421_MOESM1_ESM.docx]

**Additional File 1**

Bibliographic/literature search.

| **DATABASE** | **SEARCH STRATEGY**  **(**TITLE-ABS-KEY) | **RESULTS** |
| --- | --- | --- |
| PubMed | (elderly OR older adults OR aged OR older) AND (multiple AND health AND conditions OR multimorbid OR multimorbidity OR non-communicable disease OR NCD OR chronic disease OR chronic condition) AND (intervention OR program OR programme OR visit* programe OR home visit* OR home visiting OR home based) AND (nurses, visiting OR home visits OR nurs* students OR human volunteers OR trained volunt*) | [275](https://pubmed.ncbi.nlm.nih.gov/?term=%28elderly%5BTitle%2FAbstract%5D+OR+older+adults%5BTitle%2FAbstract%5D+OR+aged%5BMeSH+Terms%5D+OR+older%29+AND+%28multiple+AND+health+AND+conditions%5BTitle%2FAbstract%5D+OR+multimorbid%5BTitle%2FAbstract%5D+OR+multimorbidity%5BTitle%2FAbstract%5D+OR+non-communicable+disease%5BTitle%2FAbstract%5D+OR+NCD%5BTitle%2FAbstract%5D+OR+%28chronic+disease%5BMeSH+Terms%5D%29+OR+chronic+condition%29+AND+%28intervention%5BTitle%2FAbstract%5D+OR+program%5BTitle%2FAbstract%5D+OR+programme%5BTitle%2FAbstract%5D+OR+visit*+programe%5BTitle%2FAbstract%5D+OR+home+visit*%5BTitle%2FAbstract%5D+OR+home+visiting%5BTitle%2FAbstract%5D+OR+home+based+%5BTitle%2FAbstract%5D%29+AND+%28%28nurses%2C+visiting%5BMeSH+Terms%5D%29+OR+%28home+visits%5BMeSH+Terms%5D%29+OR+%28nurs*+students%5BMeSH+Terms%5D%29+OR+%28human+volunteers%5BMeSH+Terms%5D%29+OR+trained+volunt*%29&sort=pubdate&size=200) |
| Cochrane | (elderly OR "older adults" OR aged OR older) AND (multiple AND health AND conditions OR multimorbid OR multimorbidity OR "non-communicable disease" OR NCD OR "chronic disease" OR "chronic condition") AND (intervention OR program OR programme OR "visit* programe" OR "home visit*" OR "home visiting" OR "home based") AND (nurses, visiting OR "home visits" OR "nurs* students" OR "human volunteers" OR "trained volunt*") | [416](https://www.cochranelibrary.com/es/advanced-search) |
| EMBASE  (Elsevier) | (chronic) AND (aged OR elderly) AND (home OR home visit OR home based) AND (nurses visiting OR nursing students OR volunteers) | [34](https://www--sciencedirect--com.ual.debiblio.com/search?tak=%28chronic%29%20AND%20%28aged%20OR%20elderly%29%20AND%20%28home%20OR%20home%20visit%20OR%20home%20based%29%20AND%20%28nurses%20visiting%20OR%20nursing%20students%20OR%20volunteers%29) |
| CINAHL | **((elderly OR "older adults" OR aged OR older) AND (multiple AND health AND conditions OR multimorbid OR multimorbidity OR "non-communicable disease" OR NCD OR "chronic disease" OR "chronic condition") AND (intervention OR program OR programme OR "visit* programe" OR "home visit*" OR "home visiting" OR "home based") AND (nurses, visiting OR "home visits" OR "nurs* students" OR "human volunteers" OR "trained volunt*"))** | [217](https://web--s--ebscohost--com.ual.debiblio.com/ehost/resultsadvanced?vid=21&sid=b0f75b03-bfc6-4ceb-b3a1-f089fda94974%40redis&bquery=(elderly+OR+%22older+adults%22+OR+aged+OR+older)+AND+(multiple+AND+health+AND+conditions+OR+multimorbid+OR+multimorbidity+OR+%22non-communicable+disease%22+OR+NCD+OR+%22chronic+disease%22+OR+%22chronic+condition%22)+AND+(intervention+OR+program+OR+programme+OR+%22visit*+programe%22+OR+%22home+visit*%22+OR+%22home+visiting%22+OR+%22home+based%22)+AND+(nurses%2c+visiting+OR+%22home+visits%22+OR+%22nurs*+students%22+OR+%22human+volunteers%22+OR+%22trained+volunt*%22)&bdata=JmRiPWNjbSZsYW5nPWVzJnR5cGU9MSZzZWFyY2hNb2RlPVN0YW5kYXJkJnNpdGU9ZWhvc3QtbGl2ZSZzY29wZT1zaXRl) |
| Web of Science | **((elderly OR "older adults" OR aged OR older) AND (multiple AND health AND conditions OR multimorbid OR multimorbidity OR "non-communicable disease" OR NCD OR "chronic disease" OR "chronic condition") AND (intervention OR program OR programme OR "visit* programe" OR "home visit*" OR "home visiting" OR "home based") AND (nurses, visiting OR "home visits" OR "nurs* students" OR "human volunteers" OR "trained volunt*"))** | [222](https://www.webofscience.com/wos/alldb/summary/656af0e4-9a5e-4297-9517-34f7dc0969d5-197ab8a1/relevance/1) |
| SCOPUS | **((elderly OR "older adults" OR aged OR older) AND (multiple AND health AND conditions OR multimorbid OR multimorbidity OR "non-communicable disease" OR NCD OR "chronic disease" OR "chronic condition") AND (intervention OR program OR programme OR "visit* programe" OR "home visit*" OR "home visiting" OR "home based") AND (nurses, visiting OR "home visits" OR "nurs* students" OR "human volunteers" OR "trained volunt*"))** | [35](https://www.scopus.com/results/results.uri?sort=plf-f&src=s&st1=%28elderly+OR+%22older+adults%22+OR+aged+OR+older%29+AND+%28multiple+AND+health+AND+conditions+OR+multimorbid+OR+multimorbidity+OR+%22non-communicable+disease%22+OR+NCD+OR+%22chronic+disease%22+OR+%22chronic+condition%22%29+AND+%28intervention+OR+program+OR+programme+OR+%22visit*+programe%22+OR+%22home+visit*%22+OR+%22home+visiting%22+OR+%22home+based%22%29+AND+%28nurses%2cvisiting+OR+%22home+visits%22+OR+%22nurs*+students%22+OR+%22human+volunteers%22+OR+%22trained+volunt*%22%29&sid=27377631fa684201bf1c334afabae72a&sot=b&sdt=b&sl=433&s=TITLE-ABS-KEY%28%28elderly+OR+%22older+adults%22+OR+aged+OR+older%29+AND+%28multiple+AND+health+AND+conditions+OR+multimorbid+OR+multimorbidity+OR+%22non-communicable+disease%22+OR+NCD+OR+%22chronic+disease%22+OR+%22chronic+condition%22%29+AND+%28intervention+OR+program+OR+programme+OR+%22visit*+programe%22+OR+%22home+visit*%22+OR+%22home+visiting%22+OR+%22home+based%22%29+AND+%28nurses%2c+visiting+OR+%22home+visits%22+OR+%22nurs*+students%22+OR+%22human+volunteers%22+OR+%22trained+volunt*%22%29%29&origin=searchbasic&editSaveSearch=&yearFrom=Before+1960&yearTo=Present) |
| **TOTAL** | | **1199** |
