# Supplementary material for: Effects of home visiting programmes on community-dwelling older adults with chronic multimorbidity: a scoping review
Source: BMC Nurs. 2023 Aug 12;22:266. doi: 10.1186/s12912-023-01421-7 (PMC10422812; doi:10.1186/s12912-023-01421-7)
Supplement: Supplementary file 2 — Additional file 2. Flow diagram adapted from PRISMA-ScR [51]. [file 12912_2023_1421_MOESM2_ESM.docx]

Records identified from:
Citations searching (n = 0)

**Identification of studies via databases**

Records found through searching databases
(n = 1199)

**Identification of studies via other methods**

Identification

Studies sought for retrieval (n = 0)

Duplicated records deleted
(n = 203)

Screening

Records deleted as not related to the topic (title and summary)
(n = 940)

Studies assessed for eligibility (n = 0)

Full-text articles excluded (n = 52), for not fulfilling inclusion criteria:

- Non-intervention design (n = 9)
- Age (n = 7)
- Intervention (n = 22)
- Population (not chronic multimorbidity) (n = 14)

Full-text articles evaluated for eligibility
(n = 56)

Eligibility

Studies included in the narrative synthesis

(n=4)

Included

**Additional File 2**. Flow diagram adapted from PRISMA-ScR.^51^
